# Association Between Atrial Fibrillation and the Risk of Cardiovascular Mortality Among Elderly Adults With Ischemic Stroke in Northeast China: A Community-Based Prospective Study

**DOI:** 10.3389/fnagi.2022.836425

**Published:** 2022-03-01

**Authors:** Zhenwei Xia, Wei Dang, Yang Jiang, Shuang Liu, Ling Yue, Fengshuo Jia, Qun Sun, Lei Shi, Jixu Sun, Jiao Li, Hongyun Chen

**Affiliations:** ^1^Department of Cardiology, Dalian Municipal Central Hospital, Dalian, China; ^2^Department of Ultrasound, The Fourth Affiliated Hospital of China Medical University, Shenyang, China; ^3^Department of Chronic Disease, Disease Control and Prevention of Chao Yang City, Chaoyang, China; ^4^Department of Chronic Disease, Disease Control and Prevention of Liao Yang City, Liaoyang, China; ^5^Department of Chronic Disease, Disease Control and Prevention of Dan Dong City, Dandong, China

**Keywords:** atrial fibrillation, ischemic stroke, awareness, treatment, cardiovascular mortality

## Abstract

**Background:**

Elderly people are susceptible to atrial fibrillation (AF) and ischemic stroke (IS); however, less information is known about the association between AF and the risk of cardiovascular disease (CVD) mortality in elderly population with IS. We aimed to investigate the features of AF among aged people with IS and to illustrate whether AF accounted for CVD mortality.

**Methods:**

At baseline, 790 patients with IS were enrolled from the general northeast Chinese elderly population (>60 years) between September 2017 to March 2019. The prevalence, awareness, and treatment of AF in each age group were analyzed, as well as major-related cardiovascular risk factors. The population was followed until July 31, 2021, and information on CVD death was obtained.

**Results:**

A total of 25 people had AF, and the prevalence of AF in the elderly population with IS was 3.2%. The AF prevalence grew along with age from 1% (60–64 years) to 4.3% (70–74 years) and 4.2% (≥75 years), which was higher in the urban residents than in the rural residents (5.7 vs. 2.2%, *P* = 0.014). The awareness and treatment rates of patients with AF were 80 and 8%. After a median follow-up period of 3.3 years, 58 subjects died due to CVD and 5 subjects were accompanied with AF (rate 70.6/1,000 person-years). Elderly IS patients with AF had a 3.65-fold increased risk of CVD death in the fully adjusted model when compared with non-AF participants.

**Conclusion:**

The AF prevalence increased with age among the elderly population with IS. Moreover, elderly patients with IS in northeast China with AF had a higher CVD mortality. Therefore, early screening and prompt management of AF in elderly population with IS in northeast China are required.

## Introduction

Atrial fibrillation (AF) is the most frequently sustained cardiac arrhythmia worldwide. The number of patients with AF has increased over the past 20 years and will increase over the next 30 years continually (Krijthe et al., 2013; Lippi et al., 2021). The prevalence of AF is clearly age-dependent (Kirchhof et al., 2016; Alghamdi and Chan, 2017). According to the “Expert Consensus of the French Society of Geriatrics and Gerontology and the French Society of Cardiology,” 70% of patients with AF aged over 75 years (Hanon et al., 2013), as advancing age and systemic cardiovascular disease (CVD) risk factors can lead to an abnormal atrial tissue substrate, or atrial cardiopathy (Kamel et al., 2016). Electrophysiological remodeling and oxidative stress, such as diabetes, smoking, and inflammation, are also linked to an increased risk of AF (Tse et al., 2013). In China, AF is expected to affect an estimated 5.2 million men and 3.1 million women older than 60 years by 2050 (Gasparova et al., 2017).

Ischemic stroke (IS), another global public health problem, is the second leading cause of death worldwide (Jose et al., 2014) and is one of the major complications of AF (Wolf et al., 1991). Similar to AF, IS is a disease of aging, which mostly occurs in people >65 years (No authors, 2017). Therefore, the increasing average age in the current human population can lead to the growing prevalence of AF together with IS (Tofield, 2017). The risk of mortality in IS is higher (Grysiewicz et al., 2008), especially in the aged population (Rojas et al., 2007). In addition, despite relevant progress in the diagnosis and management of patients with AF, this arrhythmia is considered to be independently associated with an increased risk of mortality after adjusting for cardiovascular comorbidities (Benjamin et al., 1998). Therefore, a focus on healthcare systems to improve early screening and treatment for the elderly population with AF and IS should be emphasized.

Previous studies have reported that AF is closely related to an increased risk of all-cause and CVD mortality (Schnabel et al., 2015; Odutayo et al., 2016). However, few of the previous studies have investigated the association between AF and the risk of CVD death in elderly people with IS in northeast China. Thus, in this study, we aimed to profile the up-to-date characteristics of AF in elderly patients with IS and to further examine the risk of CVD mortality with the onset of AF in this particular population in northeast China.

## Materials and Methods

### Study Population

**FIGURE 1:**
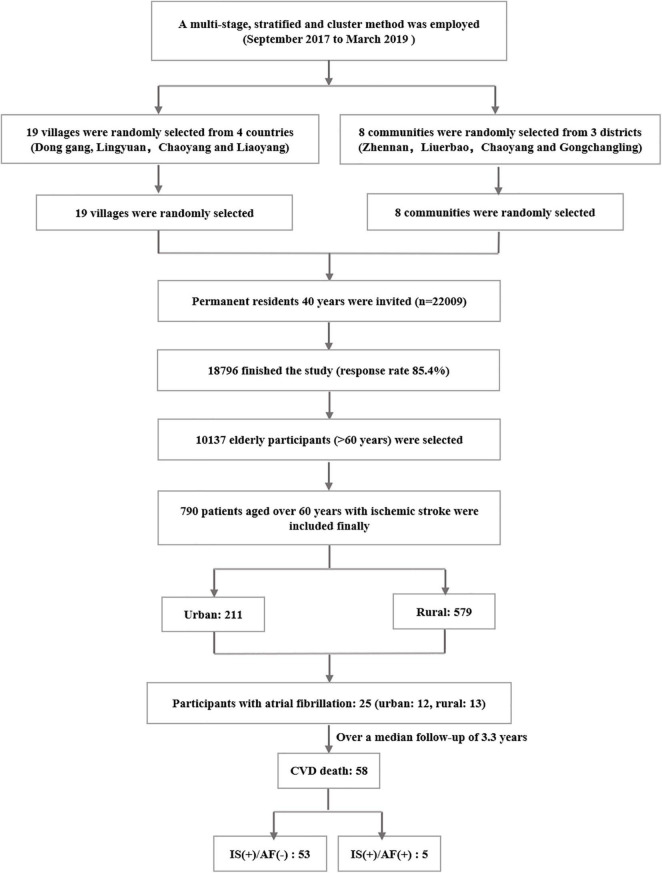
Flow chart of the patient selection process.

This is a community-based prospective cohort study. The median follow-up period is 3.33 years from September 2017 to March 2019. In total, 790 participants who aged ≥60 years with IS in the rural and urban areas of Liaoning Province in northeast China were enrolled (Figure 1). All participants were followed up for the status of survival and specific cause of death. The study was approved by the Central Ethics Committee at the China National Center for Cardiovascular Disease, Beijing, China. Written informed consent was obtained from all participants.

The clinical record, computed tomography (CT), and/or magnetic resonance imaging (MRI) during the hospital stay were required for the diagnosis of IS (Hatano, 1976). In addition, participants were confirmed for IS by well-trained neurologists according to the World Health Organization recommendations.

### Baseline Data Collection

At baseline, detailed information on demographic characteristics, lifestyle, and disease history was collected using face-to-face questionnaires, physical examinations, and laboratory tests. All investigators underwent uniform training before starting the survey. Systolic blood pressure (SBP) and diastolic blood pressure (DBP) were measured using a standardized automatic electronic sphygmomanometer. Each participant received three measurements at 2-min intervals, and the mean SBP and the mean DBP were calculated. Weight and height were measured using standardized methods, and the body mass index (BMI) (kg/m^2^), as weight divided by the square of height, was calculated. Information on current smoking, current drinking, education, annual household income, lack of exercise, and family history of hypertension was self-reported. Twelve-lead electrocardiographs (ECGs) were recorded for all participants by trained cardiologists after a 10-s rest.

Arterial fibrillation is defined by the characteristics of ECG according to the American College of Cardiology (ACC)/American Heart Association (AHA)/European Society of Cardiology (ESC) guidelines (Fuster et al., 2011). Hypertension was diagnosed using the criteria, namely, mean SBP ≥ 140 mmHg and/or mean DBP ≥ 90 mmHg, or received antihypertension medication in the past 2 weeks, according to the recommendations of the China Hypertension Guidelines 2018. Diabetes was defined as positive if the fasting plasma glucose (FPG) level was ≥7.0 mmol/L or the HbA1c level was ≥6.5%, and/or having a clinical diagnosis previously (Chamberlain et al., 2016). Dyslipidemia was defined if patients met any of the following criteria: (1) serum TC level ≥ 6.22 mmol/L, (2) serum TG level ≥ 2.27 mmol/L, (3) serum LDL-C level ≥ 4.14 mmol/L, (4) serum HDL-C level < 1.04 mmol/L, and (5) self-reported use of lipid-regulating drugs in the past 3 months (Yan et al., 2016). Overweight was determined as a BMI ranging from 24.4 to 27.9 kg/m^2^, and obesity was defined as a BMI ≥ 28.0 kg/m^2^ (Chen and Lu, 2004). Current smoking was defined as smoking more than one cigarette per day continued over 1 year, and participants who consumed alcohol more than one time per week would be considered to be currently drinking. Regular exercise was defined as moderate-intensity exercise or equivalent to walking for ≥30 min and ≥3 times per week, which moderate and heavy manual workers fulfill due to their work (Lee et al., 2017). Awareness of AF was defined as self-reporting a previous AF diagnosis. Treatment of AF was defined as a patient with AF who underwent thromboembolic prophylaxis with oral anticoagulant (OAC) therapy according to the 2012 ESC guidelines (Kirchhof et al., 2016).

### Follow-Up for Cardiovascular Disease Mortality

Cardiovascular disease-related death during follow-up was identified by the National Population Registry of the China National Statistical Office. Case ascertainment was completed until July 31, 2021 (median follow-up: 3.3 years). The cause of death was determined by reviewing the death certificates and classified according to the death code (International Classification of Diseases, 10th revision).

### Statistical Analysis

The continuous variables with normal distribution were reported as means and standard deviations (SD), and numerical data were expressed as rates. For each age group (60–64, 65–69, 70–74, and ≥75 years at baseline), AF incidence, awareness, and treatment rates were calculated. Prevalence of several main risk factors in people with AF in northeast China and awareness, treatment, and control of main chronic diseases in people mentioned earlier were also calculated.

Moreover, we examined the risk of CVD death between patients who had AF compared with subjects without AF diagnosis. Multivariable-adjusted models include three models: model 1 was unadjusted; model 2 was adjusted for age, sex, and area (urban and rural regions); and model 3 was further adjusted for age, sex, area, BMI, hypertension, diabetes, dyslipidemia, smoking and drinking status, education, income, and physical activity. Cox proportional hazard regression models were used in the evaluation of the risk of CVD death in patients who had AF and those who did not. SPSS22.0 (SPSS Inc., Chicago, IL, United States) was used for all statistical analyses; *P*-values < 0.05 were considered statistically significant.

## Results

### Baseline Data

The average age at baseline was 69.3 ± 6.2 years, and 394 (49.9%) out of the 790 participants were men. In total, 211 (26.7%) individuals were selected from the urban region. Of the participants, 91.9% had a middle-school education or lower and 50.6% were in low-socioeconomic status with an annual household income of less than 5,000 yuan. The mean SBP and DBP levels were 156.1 ± 23.0 mmHg and 88.0 ± 12.1 mmHg, respectively. In terms of statistical differences for characteristics (*P* < 0.05), annual household income less than 5,000, mean DBP, and lack of exercise were significantly different between regions; however, mean BMI of <18.5 kg/m^2^ and current smoking and drinking between genders were statistically significant. Education of primary school or lower, mean SBP, and mean BMI were significantly different in both aspects (Table 1).

**TABLE 1:** Characteristics of the study participants (≥60 years).

Characteristics	Region		Sex		Total	*P*-value for region	*P*-value for sex
		
	Urban	Rural	Men	Women			
Participant, n (%)	211 (26.7)	579 (73.3)	394 (49.9)	396 (50.1)	790 (100.0)		
Age, year	69.9 ± 6.1	69.0 ± 6.2	69.4 ± 6.5	69.1 ± 5.8	69.3 ± 6.2	0.091	0.547
60–64	21.3	28.5	26.6	26.5	26.6	0.345	0.225
65–69	32.2	27.8	30.5	27.5	29.0		
70–74	23.7	23.3	20.1	26.8	23.4		
≥75	22.7	20.4	22.8	19.2	21.0		
Education, %							
Primary school or lower	44.5	73.6	55.1	76.5	65.8	<0.001	<0.001
Middle school	39.8	21.1	33.5	18.7	26.1		
High school or above	15.6	5.4	11.4	4.8	8.1		
Income, %							
<5000	5.7	67.0	47.7	53.5	50.6	<0.001	0.119
5000–9999	15.2	19.3	18.5	17.9	18.2		
10000–19999	14.7	7.1	8.4	9.8	9.1		
≥20000	64.5	6.6	25.4	18.7	22.0		
BMI, kg/m^2^	25.3 ± 2.9	24.5 ± 3.8	24.3 ± 3.2	25.1 ± 3.9	24.5 ± 3.6	0.008	0.004
<18.5	0.9	4.0	1.8	4.6	3.2	0.001	<0.001
18.5–23.9	30.8	41.9	42.9	34.9	38.9		
24.0–27.9	51.2	36.9	42.6	38.7	40.7		
≥28.0	17.1	17.3	12.7	21.8	17.2		
Mean SBP, mmHg	146.8 ± 17.7	159.4 ± 23.8	154.2 ± 22.7	158.0 ± 23.1	156.1 ± 23.0	<0.001	0.020
Mean DBP, mmHg	85.0 ± 10.2	89.1 ± 12.6	88.8 ± 11.7	87.3 ± 12.5	88.0 ± 12.1	<0.001	0.094
Current smoking, %	19.4	26.1	40.1	8.6	24.3	0.054	<0.001
Current drinking, %	13.7	16.1	27.7	3.3	15.4	0.425	<0.001
Lack of exercise, %	14.7	37.1	31.0	31.3	31.1	<0.001	0.916

*AF, atrial fibrillation; BMI, body mass index; DBP, diastolic blood pressure; FBG, fasting blood glucose; SBP, systolic blood pressure.*

*Data are presented as mean ± standard deviation or n (%).*

*P for region: p-values between urban and rural areas.*

*P for sex: p-values between men and women.*

### Prevalence of Atrial Fibrillation and Among Elderly Population With Ischemic Stroke

Totally, 25 people were diagnosed with AF, and the overall prevalence was 3.2% at baseline. In addition, there was a significant difference between urban and rural regions. In the age group of 65–69 years, the prevalence of AF was statistically significant between the urban and rural residents, while there were no differences between men and women in each age group and overall. In women, the prevalence was relatively higher among those who aged 70–74 years (4.7%). In men, the prevalence of subjects who aged ≥ 75 years was the highest, and the percentage was 5.6%. The age-standardized prevalence of AF was 3.2% (urban: 5.1%, rural: 2.3%, men: 3.7%, and women: 2.5%) (Table 2).

**TABLE 2:** Prevalence of atrial fibrillation (AF).

Characteristics	N	Region		Sex		Total	*P* for region	*P* for sex
			
		Urban	Rural	Male	Female			
60–64	2	2.2	0.6	1.0	1.0	1.0	0.322	1.000
65–69	8	8.8	1.2	5.0	1.8	3.5	0.004	0.193
70–74	8	4.0	4.4	3.8	4.7	4.3	0.895	0.761
≥75	7	6.3	3.4	5.6	2.6	4.2	0.414	0.455
Overall	25	5.7	2.2	3.8	2.5	3.2	0.014	0.303
ASR		5.1	2.3	3.7	2.5	3.2		

*ASR, age-standardized rates by China Census Population 2010.*

### Awareness and Treatment of Atrial Fibrillation Among Elderly Population With Ischemic Stroke

**FIGURE 2:**
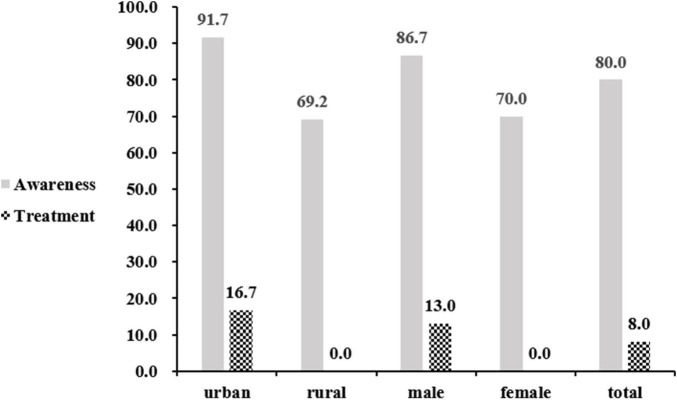
Awareness and treatment of atrial fibrillation (AF).

Among those subjects with AF, 80% were aware of their diagnosis, while only 8% (2/25) were receiving treatment. Among those who aged ≥75 years, the awareness rate was 100%. The awareness rate of AF in the urban areas was higher than that in the rural places (91.7 vs. 69.2%, *P* = 0.161); however, the treatment rate in the urban areas was significantly higher than in the rural areas (91.7 vs. 46.2%, *P* = 0.015). The awareness rate of AF in men was higher than in women (86.7 vs. 70%, *P* = 0.307), but the treatment rate in men was lower (66.7 vs. 70.0%, *P* = 0.861). Both the awareness and the treatment rate between different genders were not statistically significant (Figure 2).

### Main Risk Factors in People With Atrial Fibrillation Among Elderly Population With Ischemic Stroke

The most prevalent comorbidity that is a risk factor for AF was hypertension (84%) in total, with people living in rural places having the highest rate of hypertension (92.3%) compared with urban regions (75%). The second most prevalent comorbidity in total was overweight and obesity (72%), with 83.8, 61.5, 73.3, and 70% of prevalence in urban, rural, men, and women, respectively. Of the entire cohort, 52% of people had dyslipidemia, with 58.3, 46.2, 46.7, 60% prevalence in urban, rural, men, and women, respectively; 40% of people had diabetes; 20% of people were currently smoking; 28% of people drank alcohol; and 28% of people lacked exercise. However, there were no significant differences in all these main risk factors between men and women or between urban and rural regions. Table 3 lists the prevalence of the main risk factors in 25 people with AF in northeast China.

**TABLE 3:** Prevalence of some main risk factors in 25 people with atrial fibrillation (AF) in northeast China.

	Urban		Rural		Men		Women		Total		*P* for region	*P* for sex
					
	N	Rate	N	Rate	N	Rate	N	Rate	N	Rate		
Hypertension	9	75.0	12	92.3	13	86.7	8	80.0	21	84.0	0.238	0.656
Diabetes	3	25.0	7	53.8	6	40.0	4	40.0	10	40.0	0.141	1.000
Dyslipidaemia	7	58.3	6	46.2	7	46.7	6	60.0	13	52.0	0.543	0.513
Current smoking	3	25.0	2	15.4	4	26.7	1	10.0	5	20.0	0.548	0.307
Alcohol drinking	3	25.0	4	30.8	6	40.0	1	10.0	7	28.0	0.748	0.102
Lack of exercise	2	16.7	5	38.5	4	26.7	3	30.0	7	28.0	0.225	0.856
Overweight or obesity	10	83.3	8	61.5	11	73.3	7	70.0	18	72.0	0.225	0.856

### Awareness, Treatment, and Control of Main Chronic Diseases Among Elderly Population With Ischemic Stroke

In terms of awareness, treatment, and control of three main chronic diseases (hypertension, diabetes, and dyslipidemia) in people with AF, the proportion of awareness and treatment for hypertension was the highest, accounting for 81% and 71.4% of the total people, respectively, with a significant difference between genders (*P* = 0.007). However, only 4.8% of them received proper control of blood pressure. The percentage of patients who were aware of diabetes was 70%, and the treatment rate was 60%, while 33.3% of the conditions were under control. The percentages of dyslipidemia awareness, treatment, and control were 38.5, 30.8, and 23.1%, respectively (Table 4).

**TABLE 4:** Awareness, treatment, and control of main chronic diseases in 25 people with atrial fibrillation (AF) in northeast China.

	Urban		Rural		Men		Women		Total		*P* for region	*P* for sex
					
	N	Rate	N	Rate	N	Rate	N	Rate	N	Rate		
**Hypertension**												
Awareness	8	88.9	9	75.0	12	92.3	5	62.5	17	81.0	0.422	0.091
Treatment	8	88.9	7	58.3	12	92.3	3	37.5	15	71.4	0.125	0.007
Control	0	0.0	1	8.3	1	7.7	0	0.0	1	4.8	0.375	0.421
**Diabetes**												
Awareness	3	100.0	4	57.1	4	66.7	3	75.0	7	70.0	0.175	0.778
Treatment	3	100.0	3	42.9	3	50.0	3	75.0	6	60.0	0.091	0.429
Control	0	0.0	2	66.7	2	66.7	0	0.0	2	33.3	0.083	0.083
**Dyslipidaemia**												
Awareness	3	42.9	2	33.3	2	28.6	3	50.0	5	38.5	0.725	0.429
Treatment	2	28.6	2	33.3	2	28.6	2	33.3	4	30.8	0.853	0.853
Control	1	14.3	2	33.3	2	28.6	1	16.7	3	23.1	0.416	0.612

### The Risk of Cardiovascular Disease Death in Participants With and Without Atrial Fibrillation

**FIGURE 3:**
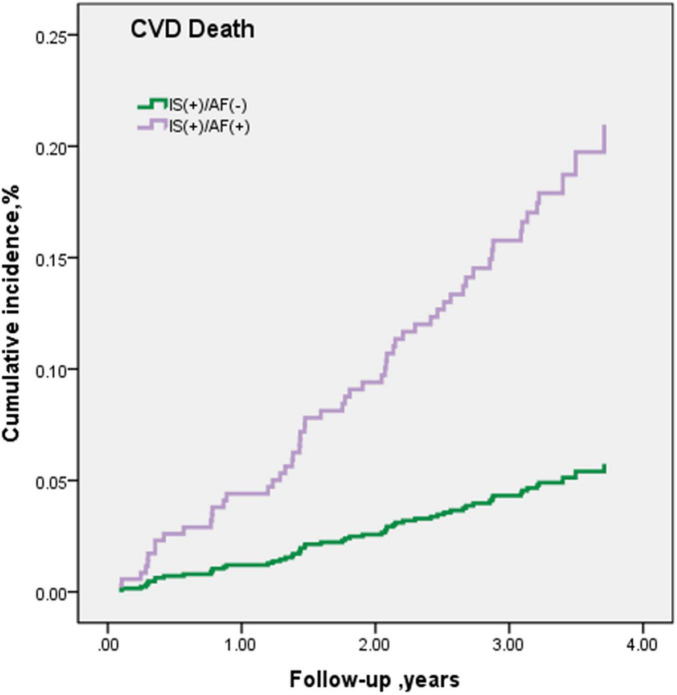
CVD death during follow-up period.

Over a median follow-up period of 3.3 years, among non-AF subjects, the risk of CVD death was 22.0/1,000 person-years (PY). The risk of CVD death with the onset of AF was 70.6/1,000 PY, which was associated with a 3.24-fold (95% CI: 1.29–8.11) risk higher than the non-AF group. After adjusting for age, sex, and area (model 2), the risk of CVD death was slightly rising to 3.26-fold (95% CI: 1.29–8.25) compared with the rate ratio of model 1. Additional adjustment for multivariable in model 3 increased the risk up to 3.65-fold (95% CI: 1.43–9.32) for CVD death (Table 5 and Figure 3). Among patients with AF receiving OAC therapy, no one died of CVD. However, in patients without OAC therapy, 21.7% (5/23) of patients died of CVD.

**TABLE 5:** Rates and rate ratios with 95% CI of CVD mortality according to the prevalence at baseline.

Category	Number of events	Follow-up (person-years)	Rate (per 1,000 person-years)	Model 1	Model 2	Model 3
**CVD death**						
IS(+)/AF(−)	53	2404.37	22.0	1	1	1
IS(+)/AF(+)	5	70.83	70.6	3.24 (1.29, 8.11)	3.26 (1.29, 8.25)	3.65 (1.43, 9.32)

*Model 1 was not adjusted; Model 2 was adjusted for age, sex, and region; and Model 3 was adjusted for age, sex, region, BMI, hypertension, diabetes, dyslipidemia, current smoking, current drinking, education, income, and physical activity.*

## Discussion

In this study, we investigated the prevalence of AF among the IS population aged from 60 to ≥75 years in northeast China and whether AF accounted for the risk of CVD mortality in this population. Compared with those living in the rural areas, elderly urban residents with IS were more likely to have AF and, the prevalence of AF was higher in men than in women. A high prevalence of comorbidities, including hypertension (84%), diabetes (40%), and dyslipidemia (52%), was observed in elderly individuals with AF and IS; however, the control rates of these chronic diseases were frustratingly low. Moreover, we observed that elderly patients with IS combined with AF had a 3.65-fold higher risk of CVD death in the fully adjusted model than the patients without AF. Those findings indicated that the elderly population with IS combined with AF tended to have worse clinical outcomes, and a particular focus on the management of elderly population with IS combined with AF should be highlighted in northeast China.

### Prevalence of Atrial Fibrillation Among Elderly Population With Ischemic Stroke

Age was considered to be the greatest risk factor for AF during the past 50 years (Schnabel et al., 2015). Consistent with one previous study (Kirchhof et al., 2016), our study illustrated a similar increasing trend of AF prevalence with advancing age with a prevalence of 1% in people aged 60–64 years, and then soared to 4.3 and 4.2% in people aged 70–74 and ≥75 years, respectively. However, in this study, the rate of AF prevalence was relatively higher among elderly population with IS than that among the general elderly population (Xing et al., 2020). In addition, it is likely due to the reason that aged patients with IS have more traditional risk factors, such as hypertension, diabetes, and dyslipidemia, and even more severe for AF.

In the elderly population with IS, AF prevalence is higher in urban areas than in rural areas. The reason for this region-related difference might be different lifestyles and environmental risk factors over time (Wang et al., 2018). Previous studies (Miyasaka et al., 2006; Schnabel et al., 2015) indicate that the age-adjusted prevalence of AF is higher in men compared with women. Although the rate of AF prevalence in this study is consistent with previous studies, we did not find a significant difference.

### Awareness and Treatment of Atrial Fibrillation

The overall awareness of AF in this study was 80%, and a significant difference was neither noted between the urban and rural areas nor between men and women. During the follow-up, the CVD death rate for patients without OAC therapy was significantly higher than that in the OAC therapy group. Therefore, the OAC therapy should be highlighted in elderly patients with IS and AF. We also found that there was a statistical significance between urban and rural treatments, and it is possibly due to the rapid construction of basic medical facilities and increasing level of cultural life in both areas, especially in the rural region so that more people can easily be aware of their health conditions, but there are still defects in rural healthcare. Besides, the education level and annual income are possibly another reason. For instance, according to baseline data in our study, 64.5% of urban people receive more than 20,000 RMB per year, while 67% of rural people can only have less than 5,000 RMB, so that the urban population can receive better medical care.

### Some Risk Factors and Main Chronic Diseases in the Atrial Fibrillation Population

Several previous studies identified some risk factors, including hypertension, diabetes, obesity, and alcohol consumption (Benjamin et al., 1994; Sun et al., 2015). In our study, we found that a large proportion of the population with AF had hypertension (84%), diabetes (40%), dyslipidemia (52%), and overweight and obesity (72%). Although people with AF had awareness of those chronic diseases and received treatments, the control rates were unsatisfactory, especially for hypertension; only 4.8% of hypertension status was under control. We also found a significant difference in hypertension treatment between men and women, indicating that women had better treatment compliance rates than men. Thus, management of main risk factors and chronic diseases is recommended in northeast China in the coming decades.

### Risk of Cardiovascular Disease Mortality Among Atrial Fibrillation Population

Increased risk of mortality is a well-known complication of AF (Magnussen et al., 2017). We investigated the association between AF and the risk of CVD mortality in this population, irrespective of the presence or absence of some main traditional risk factors. In addition, we observed that elderly patients with AF had a 3.65-fold higher risk of CVD death than their counterparts without AF in the fully adjusted model. AF is associated with abnormal blood stasis so that atrial hypo-contractility and the loss of atrial kick, atrial structural remodeling, and activation of platelets and the coagulation cascade would emerge (Kim and Roh, 2016). Therefore, AF promotes thrombus formation and cerebral embolism, which may cause or aggravate stroke. It is known that in patients with AF, a fast heart rate would occur. A previous study in 20,165 patients with IS illustrated that basal heart rate was associated with mortality (Bohm et al., 2012). Thus, for the elderly population with IS, it is pivotal to monitor their condition in cardiovascular, and timely diagnosis and intervention for AF are important to reduce the risk of death.

### Strengths and Limitations

Our study comprehensively profiled the prevalence of AF among elderly population with IS in northeast China, providing an opportunity to assess the CVD risk in this particular population accurately. However, this study has several limitations. First, we recorded the total incidence of AF without further classified AF subtypes, as well as the etiology and pathogenesis of AF, therefore further studies on the association between AF subtypes, the etiology, and pathogenesis of AF and risk of CVD mortality are needed. Second, awareness and treatment conditions were self-reported, which was inevitable to recall bias. Third, the sample size of patients with AF in this study cohort was relatively small and the follow-up period was short; however, the association between AF and the risk of CVD mortality was statistically significant. In addition, we did not further distinguish between male death and female death. Finally, as the population in this study was limited to the northeast regions of China, our results might be limited in other regions. Therefore, further studies among populations in different regions or races are needed to verify our findings.

## Conclusion

Atrial fibrillation is an important risk factor to consider when assessing older adults with IS as it is associated with an increased risk of CVD-related mortality. Our study indicates that AF was associated with an increased risk of death among the elderly people with IS, therefore, early screening and management of AF among this specific population should be highlighted.

## Data Availability Statement

The original contributions presented in the study are included in the article/supplementary material, further inquiries can be directed to the corresponding authors.

## Ethics Statement

The studies involving human participants were reviewed and approved by the Central Ethics Committee at the China National Center for Cardiovascular Disease (Beijing, China). The patients/participants provided their written informed consent to participate in this study.

## Author Contributions

HC and JL were responsible for the study designing, conducting the data, and writing the manuscript. ZX participated in the study design and conducted the study. YJ, WD, SL, LY, FJ, QS, LS, and JS were involved in data collection. All authors contributed to the protocol and approved the final manuscript.

## Conflict of Interest

The authors declare that the research was conducted in the absence of any commercial or financial relationships that could be construed as a potential conflict of interest.

## Publisher’s Note

All claims expressed in this article are solely those of the authors and do not necessarily represent those of their affiliated organizations, or those of the publisher, the editors and the reviewers. Any product that may be evaluated in this article, or claim that may be made by its manufacturer, is not guaranteed or endorsed by the publisher.
